# Real-World Management and Clinical Outcomes of Stroke Survivors With Atrial Fibrillation: A Population-Based Cohort in Spain

**DOI:** 10.3389/fphar.2021.789783

**Published:** 2021-12-13

**Authors:** Clara L. Rodríguez-Bernal, Francisco Sanchez-Saez, Daniel Bejarano-Quisoboni, Judit Riera-Arnau, Gabriel Sanfélix-Gimeno, Isabel Hurtado

**Affiliations:** ^1^ Health Services Research Unit, Foundation for the Promotion of Health and Biomedical Research of Valencia Region (FISABIO), Valencia, Spain; ^2^ Research Network on Health Services in Chronic Diseases, Red de Investigación en Servicios de Salud en Enfermedades Crónicas (REDISSEC), Valencia, Spain; ^3^ Clinical Pharmacology Service, Vall d’Hebron Barcelona Hospital Campus, Vall d’Hebron Hospital Universitari, Barcelona, Spain

**Keywords:** secondary prevention, stroke, atrial fibrillation, real-world data, treatment strategies, oral anticoagulants, antiplatelets

## Abstract

**Objective:** Despite the continuous update of clinical guidelines, little is known about the real-world management of patients with atrial fibrillation (AF) who survived a stroke. We aimed to assess patterns of therapeutic management of stroke survivors with AF and clinical outcomes using data from routine practice in a large population-based cohort.

**Methods:** A population-based retrospective cohort study of all patients with AF who survived a stroke, from January 2010 to December 2017 in the Valencia region, Spain (*n* = 10,986), was carried out. Treatment strategies and mean time to treatment initiation are described. Temporal trends are shown by the management pattern during the study period. Factors associated with each pattern (including no treatment) vs. oral anticoagulant (OAC) treatment were identified using logistic multivariate regression models. Incidence rates of clinical outcomes (mortality, stroke/TIA, GI bleeding, and ACS) were also estimated by the management pattern.

**Results:** Among stroke survivors with AF, 6% were non-treated, 23% were prescribed antiplatelets (APT), 54% were prescribed OAC, and 17% received OAC + APT at discharge. Time to treatment was 8.0 days (CI 7.6–8.4) for APT, 9.86 (CI 9.52–10.19) for OAC, and 16.47 (CI 15.86–17.09) for OAC + APT. Regarding temporal trends, management with OAC increased by 20%, with a decrease of 50% for APT during the study period. No treatment and OAC + APT remained relatively stable. The strongest predictor of no treatment and APT treatment was having the same management strategy pre-stroke. Those treated with APT had the highest rates of GI bleeding and recurrent stroke/TIA, and untreated patients showed the highest rates of mortality.

**Conclusion:** In this large population-based cohort using real-world data, nearly 30% of AF patients who suffered a stroke were untreated or treated with APT, which overall is not recommended. Treatment was started within 2 weeks as recommended, except for OAC + APT, which was started later. The strong association of APT treatment or non-treatment with the same treatment strategy before stroke occurrence suggests a strong therapeutic inertia and opposes recommendations. Patients under these two strategies had the highest rates of adverse outcomes. An inadequate prescription poses a great risk on patients with AF and stroke; thus monitoring their management is necessary and should be setting-specific.

## Introduction

Stroke is currently the second leading cause of death and an important contributor to disability-adjusted life years worldwide (Lozano et al., 2012; Murray et al., 2012). Patients who have survived a stroke are at an increased risk of a recurrent event, and recurrent strokes constitute an important proportion (25–30%) of all preventable strokes (Rothwell et al., 2004; Hata et al., 2005; Mohan et al., 2009; Sun et al., 2013; Flach et al., 2020). Atrial Fibrillation (AF), the most common cardiac arrhythmia (Steinberg and Piccini, 2014), increases the risk of stroke and is one of the leading causes of cerebrovascular mortality and morbidity (Jørgensen et al., 1996; Lin et al., 1996).

These figures offer a clear picture of the importance of secondary prevention of stroke in patients with AF. Data on routine management of patients with AF (for primary prevention of stroke) indicate that treatment is poor (Kakkar et al., 2013; Sabouret et al., 2015; Carnero Montoro et al., 2017). However, the occurrence of an adverse event (i.e., a stroke) is likely to compel physicians to modify the therapeutic management of these patients. Furthermore, relatively recent changes, such as the introduction of non-VKA oral anticoagulants (NOAC) into the market, and the development of new thromboembolic and bleeding risk scales (Lane and Lip, 2012) might have changed the management of patients with AF who have survived a stroke. Physicians have to weigh the risk/benefit of preventing the recurrence of stroke while avoiding hemorrhagic events. Additionally, timing of initiation of drug therapy is still an unresolved challenge (Klijn et al., 2019; Seiffge et al., 2019). All these factors make decision making a complex issue for physicians treating these patients. Current recommendations on secondary prevention of stroke in patients with AF suggest antiplatelet therapy in the first 48 h after ischemic stroke and to start anticoagulation therapy at day 3 or 4 from the index stroke in patients with mild stroke and small infarcts (<1.5 cm) and at day 7 for moderate infarcts. In the case of large infarcts, the recommendation is to start OAC therapy at day 14 after the index stroke (Klijn et al., 2019; Seiffge et al., 2019). Antiplatelets are not indicated beyond this initial time (Klijn et al., 2019; Seiffge et al., 2019). However, studies on the real-world management of these patients including the whole picture of all possible treatments (including no treatment) are lacking, and it is unknown if the real world meets guidelines’ recommendations.

Knowledge on patterns of management in the real world, predictors for these treatment patterns and their evolution over time, and clinical outcomes will help the design of strategies to improve the secondary prevention of stroke in these patients.

We aimed to assess patterns of management of stroke survivors with atrial fibrillation and clinical outcomes using data from routine practice in a large population-based cohort.

## Methods

### Design

A population-based retrospective cohort of all patients with a diagnosis of AF, discharged alive following an ischemic stroke or transient ischemic attack (TIA) from any Valencia Health System (VHS) hospital from January 1, 2010, to December 31, 2017, was constructed. Patients were followed for a minimum of 365 days from the date of hospital discharge (index date).

### Population and Setting

The study was set in the Valencia region and, specifically, in the population covered by the VHS, the public health system serving about 97% of the region’s population (≈5 million inhabitants) providing universal free healthcare services (except drug copayment). We identified all patients aged 18 years and over with AF (diagnosis code of International Classification of Diseases, ninth Revision, Clinical Modification [ICD-9-CM] 427.3x and 10th Revision, Clinical Modification [ICD-10-CM] I48.x), discharged alive from VHS hospitals (index date) with a main diagnosis of acute ischemic stroke or TIA ([ICD-9-CM]/[ICD-10-CM] 433.x1, 434.x1, 436.xx/I63.x, I67.81, I67.82, I67.89 for stroke and 435.x/G45.x, I67.848 for TIA) between 2010 and 2017. One year of look-back period was used to define the baseline characteristics of the population. Patients who died within 30 days after the index date were excluded due to the short timeframe to assess treatment initiation. Also, people without pharmaceutical/health coverage by VHA, mainly some government employees whose prescriptions are reimbursed by civil service mutual insurance companies and thus not included in the pharmacy databases of the VHA, and patients not registered in the municipal census (non-residents or temporary residents) were excluded because of limitations on the follow-up.

### Data Sources

Data were obtained from the Valencia Health System Integrated Database (VID), which combines data sources linking them at an individual level through a single anonymized patient identifier. The main source of data was the VHS ambulatory Electronic Medical Record (EMR), which includes information on diagnoses, personal medical history, laboratory test results, lifestyle factors, and information on both physician prescriptions and dispensations from community pharmacies. The information on hospitalizations was based on the minimum basic dataset (MBDS) at hospital discharge, a synopsis of clinical and administrative information on all hospital discharges, including diagnoses and procedures. The population information system (SIP) provides information on the population covered by the VHS and registers certain demographic characteristics, including the geographical location of each person and the dates and causes of VHS discharge, including deaths. A detailed description of the sources of data can be found elsewhere (García-Sempere et al., 2020).

### Ethics

The study was approved by the Institutional Review Board of the Public Health General Directorate of the Valencia Health Authority and the Center for Public Health Research (September 29, 2017) and by the Ethics Committee for Drug Research of the “Hospital Clínico-Universitario de Valencia” (September 5, 2018). Written informed consent for participation was not required for this study in accordance with the national legislation and the institutional requirements. All patient data were transferred to the research team anonymized and de-identified prior to analysis. The Regulatory Commission of Access to Ambulatory Care Information of the Valencia Health Authority approved the cession of this anonymized data.

### Therapeutic Management Ascertainment

Based on electronic pharmacy data, we assessed therapeutic management, examining the presence (or not) of a prescription (filled or not from the pharmacy) for the drug classes of interest (i.e., antiplatelets and oral anticoagulants). Exposure to three different strategies of antithrombotic therapy (ATT) was considered: antiplatelets (APT), oral anticoagulants (OAC), or both (OAC + APT). Medications included were as follows: 1) For antiplatelets: acetylsalicylic acid, clopidogrel, prasugrel, ticagrelor, triflusal, cilostazol, ticlopidine, and dipyridamole; and 2) For oral anticoagulants: acenocoumarol, warfarin, apixaban, dabigatran, rivaroxaban, or edoxaban. The initial treatment of the patients is assigned considering the start of 20 consecutive days of ATT (or 20 overlapping consecutive days for considering treatment with OAC + APT) within 60 days after hospital discharge from ischemic stroke (IS) or TIA.

### Clinical Outcome Measure

We established four clinical outcomes, measured during a follow-up (any time after the index date) as follows: 1) Mortality, 2) Hospitalizations for IS and TIA 3) Hospitalizations for gastrointestinal (GI) bleeding, and 4) Acute coronary syndrome (ACS). Only principal discharge diagnoses based on ICD9CM and ICD10CM (see Supplementary Table S1 for coding on clinical outcomes) were used to define endpoints. Out-of-hospital mortality was collected from the SIP system that, in turn, obtains this information from the mortality register. All outcomes were analyzed separately, and only the first event was considered. Patients were followed from the index date and until the relevant event, health system disenrollment, death, or end of study, whichever came first.

### Covariates/Baseline Characteristics

Covariates included relevant socio-demographic and clinical characteristics and measures of health service use at the time of discharge. We identified the following variables: age, gender, and country of origin; Clinical factors included the following: baseline diagnosis (AF or flutter), main diagnosis at admission (stroke or TIA), and several comorbidities including congestive heart failure, hypertension, diabetes mellitus, liver and renal disease, dementia, depression, cancer, coronary heart disease, venous thromboembolism (VTE), intracranial hemorrhage, gastrointestinal bleeding and other bleeding previous to the current admission (see Supplementary Table S1 for codes), and risk scores (CHADS2, CHA2DS2-VASC, and HAS-BLED scores). Health service use variables included preventive medication use in the last 3 months and length of stay.

### Statistical Analysis

The study population baseline characteristics are presented as means for continuous variables and frequencies for categorical variables according to each treatment strategy. Temporal trends of treatment initiation (patients starting treatment per month during the study period) were plotted according to the therapeutic strategy. The time to start of treatment was also assessed, as mean number of days from discharge.

Logistic multinomial regression models were used to assess the independent associations between treatment strategies and baseline covariates; then, we estimated the incidence of clinical outcomes and presented unadjusted event rates per 1,000 person-year along with 95% CIs separately for each outcome and for each treatment cohort.

All statistical analyses were conducted using STATA 14^®^ (StataCorp, College Station, TX, United States) and R software, and the 5% level of significance was considered.

## Results

### Cohort Characteristics

**FIGURE 1:**
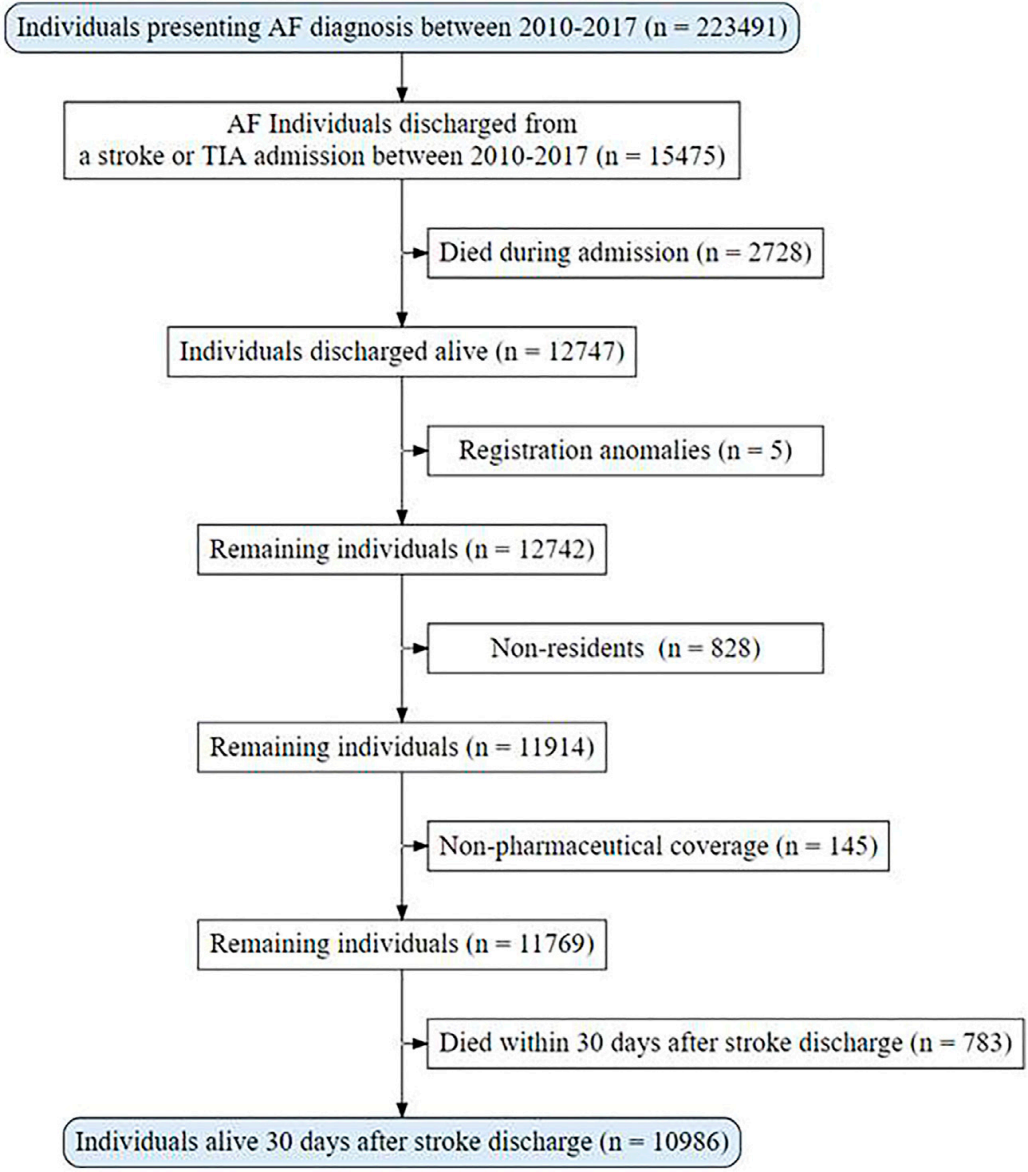
Population flowchart.

The cohort was composed of 10,986 patients with AF, discharged alive after an ischemic stroke or TIA, from which 643 (5.9%) did not receive ATT at discharge, 2,530 (23.0%) were prescribed antiplatelets (APT), 5,900 (53.7%) were prescribed OAC, and 1,913 (17.4%) received both (OAC + APT) (Figure 1).

Overall cohort characteristics and by treatment at discharge are presented in Table 1. The mean age was 78.8 years, 53% of patients were female, and 17% were foreigners. Regarding previous medication use (3 months before suffering the stroke), 34% of patients were not receiving therapy, and similar rates were observed for antiplatelet and anticoagulant use (at around 30% each) before suffering the index stroke. The more prevalent comorbidities were hypertension (75%) and diabetes (32%). The cohort had a mean CHA2DS2-VASC score of 5.58 and a mean HAS BLED score of 3.70.

**TABLE 1:** Characteristics of patients with atrial fibrillation who survived a stroke, by treatment strategy (secondary prevention).

	No treatment	APT	OAC	OAC + APT	Total
*N* (%)	643 (5.9)	2,530 (23.0)	5,900 (53.7)	1,913 (17.4)	10,986
**Age, *n* (%)**					
<65	41 (6.4)	124 (4.9)	595 (10.1)	170 (8.9)	930 (8.5)
65–74	108 (16.8)	319 (12.6)	1,316 (22.3)	480 (25.1)	2,223 (20.2)
75–84	265 (41.2)	982 (38.8)	2,752 (46.6)	915 (47.8)	4,914 (44.7)
≥ 85	229 (35.6)	1,105 (43.7)	1,237 (21.0)	348 (18.2)	2,919 (26.6)
Mean (SD)	80.7 (9.1)	82.2 (8.9)	77.6 (9.2)	77.5 (8.6)	78.8 (9.3)
**Sex**					
Female; *n* (%)	388 (60.3)	1,470 (58.1)	3,151 (53.4)	848 (44.3)	5,857 (53.3)
Country, *n* (%)					
Spain	536 (83.4)	2,163 (85.5)	4,863 (82.4)	1,561 (81.6)	9,123 (83.0)
European	42 (6.5)	105 (4.2)	250 (4.2)	114 (6.0)	511 (4.7)
Other	8 (1.2)	33 (1.3)	69 (1.2)	23 (1.2)	133 (1.2)
Unknown	57 (8.9)	229 (9.1)	718 (12.2)	215 (11.2)	1,219 (11.1)
**Diagnosis; *n* (%)**					
Atrial Flutter	20 (3.1)	99 (3.9)	134 (2.3)	46 (2.4)	299 (2.7)
Atrial Fibrillation	623 (96.9)	2,431 (96.1)	5,766 (97.7)	1,867 (97.6)	10,687 (97.3)
**Index event, *n* (%)**					
TIA	112 (17.4)	467 (18.5)	1,179 (20.0)	386 (20.2)	2,144 (19.5)
Ischemic stroke	531 (82.6)	2063 (81.5)	4,721 (80.0)	1,527 (79.8)	8,842 (80.5)
Length of stay; mean (SD)	26.9 (50.9)	16.3 (33.5)	15.2 (30.3)	10.2 (19.1)	15.3 (31.3)
**Treatment in the 3 months previous to the index date, *n* (%)**					
No treatment	365 (56.8)	851 (33.6)	2,179 (36.9)	325 (17.0)	3,720 (33.9)
APT	107 (16.6)	1,521 (60.1)	887 (15.0)	835 (43.6)	3,350 (30.5)
OAC	155 (24.1)	95 (3.8)	2,604 (44.1)	390 (20.4)	3,244 (29.5)
OAC + APT	16 (2.5)	63 (2.5)	230 (3.9)	363 (19.0)	672 (6.1)
**Comorbidities and clinical history, *n* (%)**					
CHF	144 (22.4)	418 (16.5)	902 (15.3)	356 (18.6)	1,820 (16.6)
Hypertension	492 (76.5)	1,933 (76.4)	4,358 (73.9)	1,496 (78.2)	8,279 (75.4)
Diabetes	204 (31.7)	834 (33.0)	1,690 (28.6)	741 (38.7)	3,469 (31.6)
Liver disease	21 (3.3)	45 (1.8)	67 (1.1)	30 (1.6)	163 (1.5)
Renal disease	130 (20.2)	506 (20.0)	763 (12.9)	296 (15.5)	1,695 (15.4)
Dementia	115 (17.9)	516 (20.4)	595 (10.1)	201 (10.5)	1,427 (13.0)
Depression	43 (6.7)	156 (6.2)	297 (5.0)	91 (4.8)	587 (5.3)
Malignancy	89 (13.8)	175 (6.9)	243 (4.1)	98 (5.1)	605 (5.5)
Alcohol	20 (3.1)	85 (3.4)	214 (3.6)	80 (4.2)	399 (3.6)
VTE	52 (8.1)	199 (7.9)	266 (4.5)	121 (6.3)	638 (5.8)
Coronary heart disease	76 (11.8)	458 (18.1)	617 (10.5)	559 (29.2)	1,710 (15.6)
Hemorrhagic stroke	61 (9.5)	106 (4.2)	197 (3.3)	56 (2.9)	420 (3.8)
Gastrointestinal bleeding	37 (5.8)	68 (2.7)	82 (1.4)	27 (1.4)	214 (1.9)
Other bleeding	115 (17.9)	357 (14.1)	1,012 (17.2)	356 (18.6)	1,840 (16.7)
**Baseline risk of stroke (CHADS2 and CHA2DS2-VASC) and bleeding (HAS-BLED), mean (SD)**					
CHADS2 Score	4.1 (0.9)	4.1 (0.9)	3.8 (1.0)	4.0 (1.0)	3.9 (1.0)
CHADS2-VASC score	5.7 (1.3)	5.8 (1.2)	5.4 (1.3)	5.7 (1.3)	5.6 (1.3)
HAS-BLED Score	3.6 (1.0)	4.2 (0.9)	3.4 (1.0)	4.1 (0.9)	3.7 (1.0)

TIA, transient ischemic attack; APT, antiplatelet therapy; OAC, oral anticoagulants (both VKA and NOAC); CHF, congestive heart failure; VTE, venous and pulmonary thromboembolism.

Patients not treated at discharge were mainly women (60%), more likely to be non-treated previously and had more prevalence of hemorrhagic stroke and GI bleeding. Patients prescribed antiplatelets were the eldest (mean age 82.2 years old) and were less likely to be on previous OAC treatment but had higher proportions of comorbidities such as hypertension, dementia, coronary heart disease, and had higher risk scores. Those treated with OAC were more likely to be treated with OAC previously, had a lower prevalence of comorbidities, and had the lowest mean risk scores. Patients prescribed OAC + APT were less likely to be female; their most common previous prescription was antiplatelets and were less likely to have depression, dementia, VTE, and any bleeding. They also had the shortest mean in-hospital stay. Both, patients prescribed OAC and those prescribed OAC + APT were younger, as compared to the other subgroups (mean age 77.6 and 77.5 years old, respectively) (Table 1).

### Temporal Trends

**FIGURE 2:**
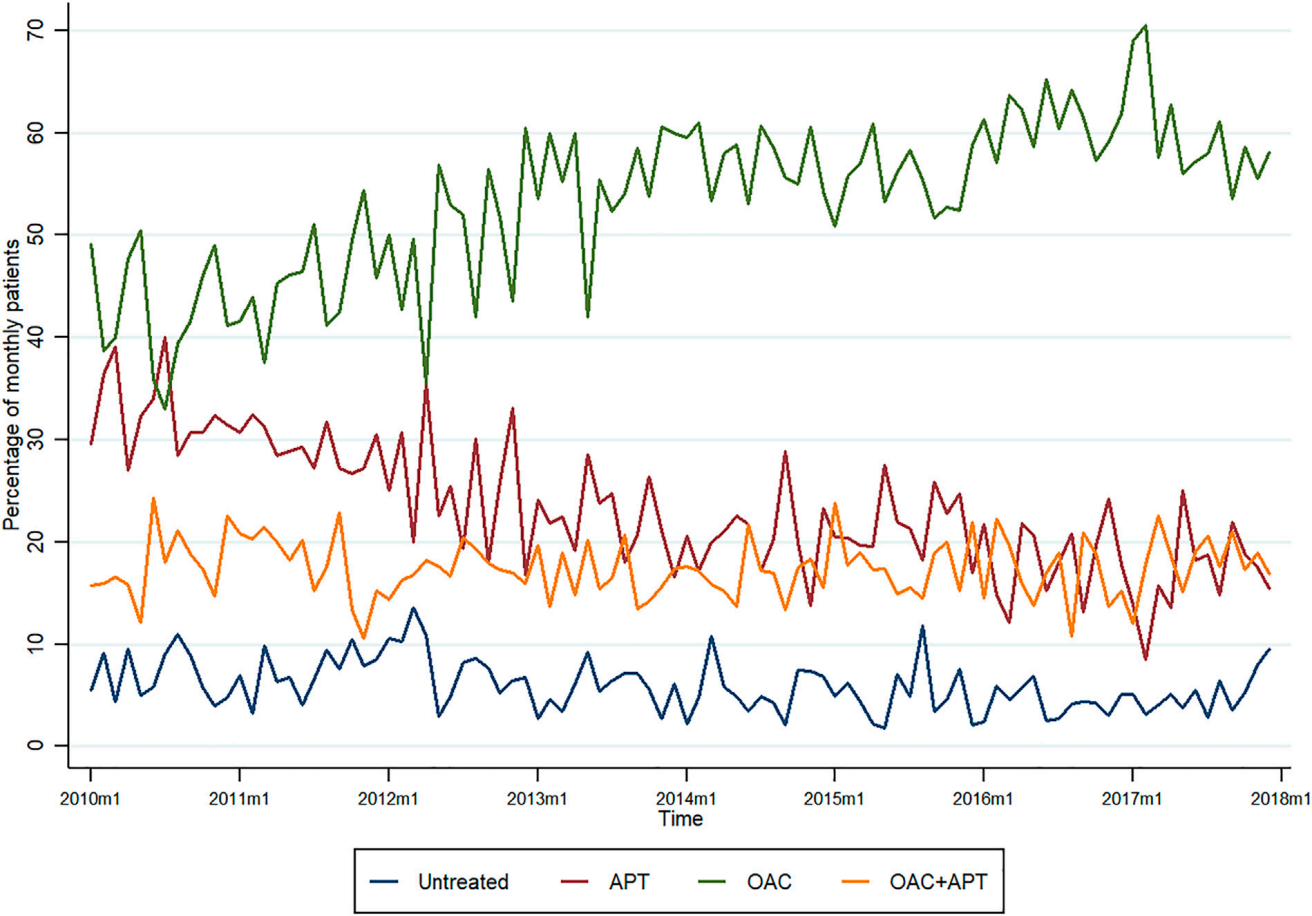
Temporal trends of treatment strategies in stroke survivors with atrial fibrillation.

Figure 2 shows treatment patterns over time for the study period (2010–2018).

At the beginning of the study period, the proportion of use did not differ much between OAC and APT (ranging from 30 to 40% for APT and from 40 to 50% for OAC), with the percentages of patients with no treatment and those treated with OAC + APT, being considerably lower.

The trend for untreated patients was relatively stable (5–10%) along the study period (although fluctuations are observed). A similar pattern was observed for OAC + APT, ranging from 15 to 20% most of the period.

Overall, there was an increase in the percentage of patients prescribed oral anticoagulants (from nearly 50% at the beginning to just under 60% at the end of the study period), reaching the lowest proportion in mid-2012 (35%) and a peak in 2017 (around 70%), whereas an important decrease in the prescription of antiplatelets is observed (from around 30–15%). The “breaking point” being 2012, when a steep rise is observed for OAC and an important reduction (halving the proportion of prescriptions) is observed for APT over time (Figure 2).

### Time to Treatment Initiation

Table 2 shows mean time (in days) to treatment initiation from index date, by treatment strategy. Patients treated with APT had the shortest time to start of treatment (8.02; CI: 7.59–8.45), followed by OAC patients (9.86; CI: 9.52–10.19). Patients managed with OAC + APT had the longest time to treatment (16.47; 15.86–17.09), doubling that of APT patients.

**TABLE 2:** Time to treatment initiation from discharge^a^ in a cohort of patients with atrial fibrillation who survived a stroke, by treatment strategy (secondary prevention).

	*N*	Mean	SD	95% IC
APT	2,530	8.0	0.2	7.6–8.4
OAC	5,900	9.9	0.2	9.5–10.2
OAC + APT	1913	16.5	0.3	15.9–17.1

Mean time (in days) to treatment initiation from discharge.

a These figures concern patients surviving 30 days after hospital discharge (that is, patients finally included in the cohort).

APT, antiplatelet; OAC, oral anticoagulants.

### Predictors of Treatment Strategy

**FIGURE 3:**
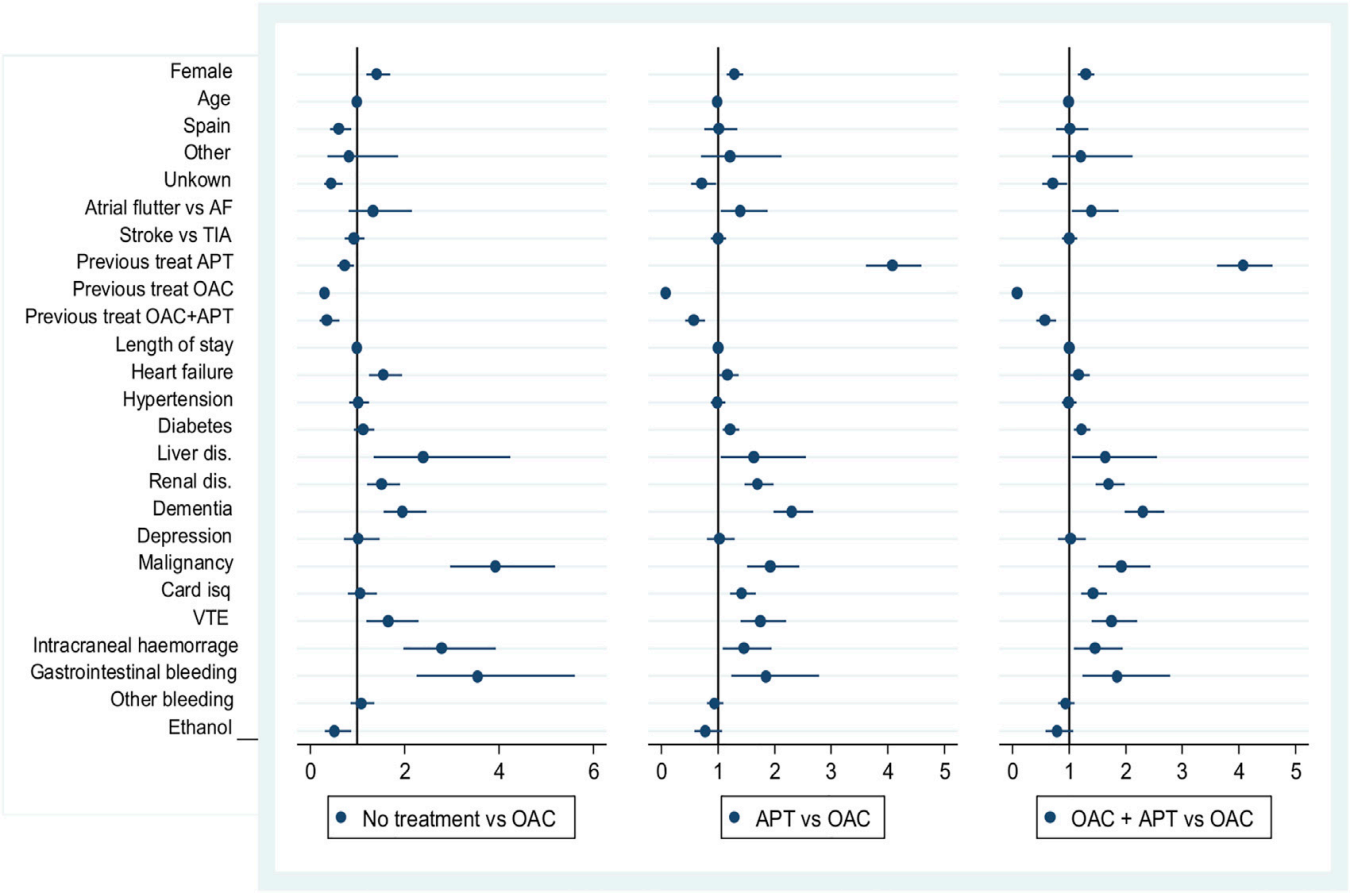
Predictors of treatment strategies in stroke survivors with atrial fibrillation.

In the adjusted analysis, compared to OAC treatment, no treatment antiplatelet treatment post-stroke were positively associated with female sex and certain comorbidities such as congestive heart failure, liver or renal disease, dementia, cancer and VTE, and previous bleeding (IC hemorrhage and GI bleeding). However, the strongest predictors were previous treatment being the same as the current treatment strategy. See Figure 3 and Supplementary Table S2.

Predictors of the combined OAC + APT prescription were previous use of the same treatment strategy (OR 7.56, 95% CI: 6.11–9.35) or previous antiplatelet use (OR 5.3, 95% CI: 4.54–6.19) and to a lesser extent diabetes, liver disease, coronary heart disease, and VTE.

### Clinical Outcomes

Incident rates of the clinical outcomes assessed are shown in Table 3.

**TABLE 3:** Incidence rates of clinical outcomes in patients with atrial fibrillation who survived a stroke, by treatment strategy.

Outcome	Events	Person-year	Rate	95% CI
**Non-treated (*n* = 643)**				
GI Bleeding	17	1,503.5	11.3	7.0–18.2
ACS	11	1,537.4	7.1	4.0–12.9
Stroke or TIA	84	1,443.1	58.2	47.0–72.1
Death	431	1,545.8	278.8	253.7–306.4
**APT (*n* = 2,530)**				
GI Bleeding	100	6,456.2	15.5	12.7–18.8
ACS	64	6,512.8	9.8	7.7–12.5
Stroke or TIA	366	6,001.7	61.0	55.0–67.6
Death	1,684	6,618.2	254.4	242.6–266.9
**OAC (*n* = 5,900)**				
GI Bleeding	215	19,823.5	10.8	9.5–12.4
ACS	144	19,873.5	7.2	6.1–8.5
Stroke or TIA	639	18,968.4	33.7	31.2–36.4
Death	2,311	20,148.1	114.7	110.1–119.5
**OAC + APT (*n* = 1,913)**				
GI Bleeding	88	6,504.4	13.5	11.0–16.7
ACS	97	6,487.8	14.9	12.2–18.2
Stroke or TIA	274	6,069.5	45.1	40.1–50.8
Death	833	6,674.3	124.8	116.6–133.6
**All Patients (*n* = 10,986)**				
GI Bleeding	420	34,287.7	12.2	11.1–13.5
ACS	316	34,411.6	9.2	8.2–10.2
Stroke or TIA	1,363	32,482.8	42.0	39.8–44.2
Death	5,259	34,986.5	150.3	146.3–154.4

GI bleeding, gastrointestinal bleeding; ACS, acute coronary syndrome; TIA, transient ischemic attack; APT, antiplatelet therapy; OAC, oral anticoagulants (both VKA and NOAC); CI, confidence interval.

### GastroIntestinal Bleeding

Incident rates of GI bleeding for the whole population were 12.25 per 1,000 person-years (95% CI: 11.13–13.48), with the highest rates seen for those treated with APT (15.49, 95% CI: 12.73–18.84) and the lowest rates seen for OAC-treated patients (10.48, 95% CI: 9.49–12.39).

### Acute Coronary Syndrome

Regarding ACS, incident rates for the whole population were 9.18 per 1,000 person-years (95% CI: 8.22–10.22). Patients treated with OAC + APT showed the highest rates of ACS (14.95, 95% CI: 12.25–18.24), whereas those untreated showed the lowest rates (7.15, 95% CI: 3.96–12.92).

### Recurrent Stroke/Transient Ischemic Attack

Incident rates for recurrent stroke were 41.96 per 1,000 person-years (95% CI: 39.79–44.25) overall, with the highest rates seen for those treated with APT (60.98, 95% CI: 55.04–67.56) and the lowest rates seen for patients treated with OAC (33.69, 95% CI: 31.17–36.40).

### Death

Regarding mortality, incident rates were 150.31 per 1,000 person-years (95% CI: 146.3–154.4) overall. Patients not receiving treatment showed the highest rates of mortality (278.82, 95% CI: 253.7–306.4) whereas those treated with OAC showed the lowest rates (114.7, 95% CI: 110.1–119.5).

## Discussion

Despite the existence of clinical guidelines and given the complexity of decision-making physicians face with these patients, the real-life management of people with AF who have survived a stroke and the extent to which recommendations are met are largely unknown to date. We identified a large population-based cohort of patients with AF, discharged alive after a stroke, and found four treatment strategies adopted by their physicians: no treatment (6%), antiplatelet treatment (23%), OAC treatment (54%), and OAC + antiplatelets (17%), meaning that–at least-about one-third of the patients with AF did not receive the recommended therapy after suffering a stroke. Regarding temporal trends in the treatment strategies, overall, an increase in the case of OAC and reduction in the case of APT is observed, especially from 2012 onwards. Prescription trends in these two treatments behave like a “mirror” with increases in use of one of them corresponding with decreases in use of the other, suggesting that a high proportion of those patients treated with antiplatelets at the beginning of the study period are being treated with OAC at the end. No treatment and OAC + APT treatment remained relatively stable along the study period, although slight fluctuations were observed. Time to treatment initiation ranged between 8 days for APT patients and 16 days for OAC + APT patients.

The most important predictors of no treatment or antiplatelet treatment (vs. OAC) were having previously the same treatment strategy, previous bleeding, female sex, and certain comorbidities. Regarding OAC + APT treatment, the strongest predictors were the previous use of the same treatment strategy or antiplatelets and to a lesser extent diabetes, liver disease, ischemic heart disease, and VTE.

Regarding clinical outcomes, we found that patients treated with OAC (the recommended therapy, overall) had the lowest rates of GI bleeding, recurrent stroke/TIA, and mortality, whereas those treated with APT had the highest rates of GI bleeding and recurrent stroke/TIA. Those treated with OAC + APT showed the highest rates of ACS, and untreated patients showed the highest rates of mortality (followed by those treated with APT).

### Comparison With Existing Evidence

Guidelines recommend long-term OAC treatment for the secondary prevention of stroke in patients with AF (Kernan et al., 2014; Klijn et al., 2019) and OAC in combination with antiplatelet treatment—mainly P2Y_12_ inhibitors—in the case of acute coronary syndrome co-existence (Klijn et al., 2019; Hindricks et al., 2021). Therefore, the finding of 6% of patients receiving no treatment at all and 23% receiving only antiplatelets after suffering a stroke shows a huge room for improvement in the management of these patients. It is true that when analyzing the time trends of routine care, we observed a decrease in the prescription of antiplatelet treatment, but still around 15% of patients were treated with these drugs at the end of the study period (2018). The great increase in the use of OAC and corresponding decrease in that of APT from 2012 onwards, might be a consequence of diverse factors: 1) the introduction into the market of NOAC drugs, which happened at the end of 2011 in Spain and grew rapidly as a safe-and in many cases was seen as a more convenient-alternative to VKA, increasing the initial OAC prescription in patients with AF both in Spain (Rodríguez-Bernal et al., 2017) and overseas (Desai et al., 2014). 2) The change in recommendations for management of these patients, with USA guidelines explicitly stating in 2014 that antiplatelet therapy cannot be recommended for stroke prevention in AF patients (Kernan et al., 2014), a recommendation adopted later on by European guidelines, with OAC therapy recommended over no treatment or antiplatelet treatment for secondary stroke prevention (Klijn et al., 2019).

In regards to time to treatment initiation (not taking into account the therapeutic strategy used), it was within the recommended days 3–14, except for OAC + APT, which was started later. These figures concern patients surviving 30 days after hospital discharge (that is, patients finally included in the cohort). It has been shown that patients treated with OAC after 14 days, have a higher risk of an ischemic event or bleeding (Paciaroni et al., 2015). It could be assumed that it applies also for combined therapy.

Regarding predictors of treatment strategy in the real world, we found that the most important predictors of no treatment or antiplatelet treatment (vs. OAC) were having previously the same treatment strategy, previous bleeding, female sex, and certain comorbidities. Keeping AF patients untreated or treated with APT even after suffering a stroke, suggests a strong therapeutic inertia (Khunti and Davies, 2017) for the management of stroke survivors with AF. The fact that previous bleeding was also a strong determinant shows the reluctance of physicians to prescribe OAC to patients with a high or moderate bleeding risk. However, it has been shown that APT has a similar (or even higher in the elderly) bleeding risk than OAC, but an inferior profile in avoiding stroke events (Mant et al., 2007; Lip, 2011). Regarding female sex as a determinant for no treatment or APT treatment, this team of researchers finds no reasonable explanation for this association.

Stroke occurrence and age are the most important risk factors for recurrence of stroke (Hindricks et al., 2021); therefore, it is likely that an important percentage of these patients suffer a preventable undesired event as a consequence of the undertreatment. In fact, when assessing the association of each management strategy with clinical outcomes, we found that patients treated with APT had the highest rates of GI bleeding and recurrent stroke/TIA, whereas untreated patients showed the highest rates of mortality. On the other hand, patients treated with OAC had the lowest rates of GI bleeding, recurrent stroke/TIA, and mortality. These results are in line with recommendations on OAC treatment as the first line therapy in prevention of stroke recurrence.

### Strengths and Limitations

Our study has reliable data on clinical characteristics including diagnoses and procedures, health service utilization, and prescription and dispensing, and individual-level data on sociodemographics, retrieved through the linkage of several electronic databases including EMR, which is an advantage over studies that base their data on administrative claims. Besides, our cohort is population-based. It uses data of the population covered by the public health system, which virtually covers the totality of the inhabitants of the region. The most important limitation of our study is that information biases due to absent registration, or differing data recording practices in the EMR might exist, although this is an inherent problem of any study using data from routine clinical practice. Moreover, misclassification (on exposure and covariates) is expected to be non-differential across groups of study subjects. Additionally, when constructing the CHADS risk score, INR was not included, given the unavailability of data for INR values. However, it is unlikely that scores have been affected to the extent of misclassifying patients along the score ranges. Finally, we present clinical outcome incidence in a descriptive manner, so no associations or causal relationships can be inferred.

## Conclusion

In this large population-based cohort using real-world data, nearly 30% of AF patients who suffered a stroke received no treatment or were treated with APT, which overall is not recommended. Treatment was started within 2 weeks as recommended, except for OAC + APT, which was started later. The strong association of APT treatment or no treatment with the same management before stroke occurrence suggests a strong therapeutic inertia and opposes recommendations. These two management strategies were associated with the highest rates of most adverse outcomes assessed. An inadequate prescription poses a great risk on patients with AF and stroke; thus, monitoring their management is necessary and should be setting-specific.

## Data Availability

The datasets presented in this article are not readily available because we have no permission to make generated datasets available. We have been granted access to data by the Valencia Health Department, so we could make the analysis for this study but are not allowed to share it. Requests to access the datasets should be directed to Unit of Analysis of Health Information Systems (Servicio de Análisis De Sistemas de Información Sanitaria). Valencia Health Department (www.san.gva.es).
