# Supplementary material for: Real-World Management and Clinical Outcomes of Stroke Survivors With Atrial Fibrillation: A Population-Based Cohort in Spain
Source: Front Pharmacol. 2021 Dec 13;12:789783. doi: 10.3389/fphar.2021.789783 (PMC8710773; doi:10.3389/fphar.2021.789783)
Supplement: Supplementary file 1 [file DataSheet1.docx]

Supplementary Material

# Supplementary Tables

| **Table S1. International Classification of Disease, 9th and 10th edition, Clinical Modification (ICD-9-CM) codes used to define study clinical outcomes and comorbidities.** | | |
| --- | --- | --- |
| **Clinical outcomes** | **ICD-9-CM codes** | **ICD-10 codes** |
| Isquemic stroke | 433.x1, 434.x1, 436.xx | I63.x, I67.81, I67.82, I67.89 |
| TIA | 435.xx | G45, I67.848 |
| GI bleeding | 456.0, 456.20, 530.21, 530.7, 530.82, 531.00, 531.01, 531.20, 531.21,531.40, 531.41, 531.60, 531.61, 532.00, 532.01, 532.20, 532.21,532.40, 532.41, 532.60, 532.61, 533.00, 533.01, 533.20, 533.21, 533.40, 533.41, 533.60, 533.61, 534.00, 534.01, 534.20, 534.21, 534.40, 534.41, 534.60, 534.61, 535.01, 535.11, 535.21, 535.31, 535.41,535.51, 535.61, 537.83, 537.84, 562.02, 562.03, 562.12, 562.13,568.81, 569.3, 569.85, 569.86, 578.0, 578.1, 578.9 | I85.11, K22.11, K22.6, K22.8, K25.0, K25.2, K25.4, K25.6, K26.0, K26.2, K26.4, K26.6, K27.0, K27.2, K27.4, K27.6, K28.0, K28.2, K28.4,K28.6, K29.01, K29.41, K29.51, K29.61, K29.21, K29.71, K29.91, K29.81,K31.811, K31.82, K57.01, K57.11, K57.13, K57.41, K57.51, K57.53,K57.81, K57.93, K57.21, K57.31, K57.91, K57.33, K66.1, K62.5, K55.21,K63.81, K92.0, K92.1, K92.2 |
| Intracranial haemorrhage | 430.xx, 431.xx, 432.xx, 852.0x, 852.2x, 852.4x, 853.0x | I60.x, I61.x, I62.0x, I62.1,I62.9, S06.36x, S06.4x, S06.5x,S06.6x |
| Acute Coronary Syndrome | 410.00,410.01,410.10,410.11,410.20,410.21,410.31, 410.40,410.41,410.51,410.61,410.70,410.71,410.80, 410.81,410.90,410.91,411 | I20,I21 |
| **Comorbidity** | **ICD-9-CM codes** | **ICD-10 codes** |
| Congestive heart failure | 428.xx, 398.91, 402.01, 402.11, 402.91, 404.01, 404.11, 404.03, 404.13, 404.91, 404.93, 425.4 | I11.0, I13.0, I13.2, I42.0, I50.x |
| Hypertension | 401.xx - 405.xx, 437.2 | I10.x- I13.x, I15.x, I67.4 |
| Diabetes | 249.xx, 250.xx, | E08.x- E11.x, E13.x |
| Liver disease | 070.0, 070.2x, 070.4x, 070.6x, 070.71, 570.xx, 571.xx, 572.xx | B150.x,B160.x, B162.x, B190.x, K704.x, K72.x ,K766.x, I85.x |
| Renal disease | 403.x,404.x, 580.x- 586.x, 590.x,753.x | I12.x,I13.x, N00.x- N05.x, N07.x, N11.x, N14.x, N17.x-N19.x,Q61.x |
| Dementia | 290.xx, 294.xx, 330.xx, 331.xx | F00.x,F01.x, F02.x, F03.x, G30.x, G31.x |
| Depression | 296.2x, 296.3x, 298.0x, 300.4x, 301.12, 311.xx | F32.x, F33.x, F34.1 |
| Malignancy | 140.xx-208.xx | C00.x -C96.x |
| Alcohol | 291.xx, 303.xx, 305.0x, 980.0, 357.5 | E52.x, F10.x, G31.2, G62.1, G72.1, I42.6, K29.2, K70.x, K86.0, O35.4, T51.x, Z71.4 |
| VTE | 451.xx,452.xx,453.5,453.8,453.9,415.1,437.6, 453.40,453.41,453.42,434.01 | I80.x,I81.x,I82.x,I26.x,I63.6,I67.6 |
| Coronary heart disease | 410.xx-414.xx | I20.x- I25.x |
| Vascular disease | 410.xx- 414.xx, 440.xx, 443.9 | I20.x- I25.x, I70.x, I71.x, I73.9 |
| Hemorrhagic stroke | 430.xx- 432.xx, 852.0x, 852.2x, 852.4x, 853.x | I60.x, I61.x, I62.0x, I62.1, I62.9, S06.36x, S06.4x, S06.5x, S06.6x |
| GI bleeding | 456.0, 456.20, 530.21, 530.7, 530.82, 531.00, 531.01, 531.20, 531.21,531.40, 531.41, 531.60, 531.61, 532.00, 532.01, 532.20, 532.21,532.40, 532.41, 532.60, 532.61, 533.00, 533.01, 533.20, 533.21, 533.40, 533.41, 533.60, 533.61, 534.00, 534.01, 534.20, 534.21, 534.40, 534.41, 534.60, 534.61, 535.01, 535.11, 535.21, 535.31, 535.41,535.51, 535.61, 537.83, 537.84, 562.02, 562.03, 562.12, 562.13,568.81, 569.3, 569.85, 569.86, 578.0, 578.1, 578.9 | I85.11, K22.11, K22.6, K22.8, K25.0, K25.2, K25.4, K25.6, K26.0, K26.2, K26.4, K26.6, K27.0, K27.2, K27.4, K27.6, K28.0, K28.2, K28.4,K28.6, K29.01, K29.41, K29.51, K29.61, K29.21, K29.71, K29.91, K29.81,K31.811, K31.82, K57.01, K57.11, K57.13, K57.41, K57.51, K57.53,K57.81, K57.93, K57.21, K57.31, K57.91, K57.33, K66.1, K62.5, K55.21,K63.81, K92.0, K92.1, K92.2 |
| Other bleeding | 078.6, 246.3, 285.1, 286.5, 336.1, 388.69, 360.43, 362.43, 362.81, 363.61, 363.62, 363.72, 364.41, 372.72, 376.32, 377.42, 379.23, 423.0, 459.0, 599.7, 599.70, 599.71, 602.1, 621.4, 626.2, 626.5, 626.7, 626.8, 626.9, 640.8x, 641.8x, 641.9x, 641.3x, 641.1x, 666.1x, 719.1x, 782.7, 784.7, 784.8, 866.01, 866.11, 790.01, 958.2, 998.1, 998.11, 998.12 | A98.5, D62, D68.312, D68.318, D68.32, G95.19, H92.2x, H44.81x, H35.73x, H35.6x, H31.30x, H31.31x, H31.41x, H21.0x, H11.3x, H05.23x, H47.02x, H43.1x, I31.2, R58, R31.9, R31.0, N42.1, N85.7, N92.0, N92.3, N93.0, N93.8, N92.6, N93.9, O20.8x, O46.x, O72.x, M25.0x, R23.3, R04.0, R04.1, S37.019, S37.029, S37.019, S37.029, R71.0, T79.2x, D78.2x, E36.0x, E89.1x, G97.3x, G97.5x, H59.1x, H59.3x, H95.2x, H95.4x, I97.4x, I97.6x, J95.6x, J95.83x, K91.6x, K91.84x, L76.0x, L76.2x, M96.8x, N99.6x, N99.82x |
| ICD-9-CM: International Classification of Diseases, 9th Revision, Clinical Modification; ICD-10-CM: International Classification of Diseases, 10th Revision, Clinical Modification; GI: gastrointestinal VTE: venous and pulmonary thromboembolism. | | |

| **Table S2. Factors associated with each treatment strategy (Vs OAC treatment). Multinomial logistic regression analysis.** | | | | | | |
| --- | --- | --- | --- | --- | --- | --- |
|  | No treatment | | APT | | OAC+APT | |
|  | OR | 95% IC | OR | 95% IC | OR | 95% IC |
| Age; mean (SD) | 1.00 | 0.99-1.01 | 0.99 | 0.99-0.99 | 0.99 | 0.99-1.00 |
| Female | 1.42 | 1.19-1.70 | 1.29 | 1.16-1.44 | 0.81 | 0.72-0.91 |
| **Country** |  |  |  |  |  |  |
| Spain | 0.61 | 0.43-0.87 | 1.01 | 0.77-1.34 | 0.65 | 0.50-0.86 |
| Other | 0.83 | 0.37-1.86 | 1.22 | 0.70-2.11 | 0.76 | 0.43-1.35 |
| Unkown | 0.46 | 0.30-0.68 | 0.71 | 0.53-0.97 | 0.65 | 0.48-0.87 |
| **Diagnoses** |  |  |  |  |  |  |
| Atrial flutter vs AF | 1.33 | 0.81-2.15 | 1.40 | 1.05-1.87 | 0.93 | 0.64-1.33 |
| Stroke vs TIA | 0.92 | 0.73-1.15 | 0.99 | 0.87-1.15 | 1.07 | 0.93-1.24 |
| **Medication Use** |  |  |  |  |  |  |
| APT | 0.73 | 0.58-0.92 | 4.07 | 3.61-4.59 | 5.3 | 4.54-6.19 |
| OAC | 0.31 | 0.25-0.39 | 0.08 | 0.06-0.10 | 0.87 | 0.74-1.03 |
| OAC+APT | 0.35 | 0.21-0.61 | 0.57 | 0.42-0.77 | 7.56 | 6.11-9.35 |
| **Comorbidities** |  |  |  |  |  |  |
| Congestive heart failure | 1.56 | 1.25-1.94 | 1.72 | 1.01-1.36 | 1.15 | 0.98-1.33 |
| Hypertension | 1.02 | 0.83-1.25 | 0.99 | 0.88-1.30 | 1.09 | 0.95-1.25 |
| Diabetes | 1.12 | 0.93-1.36 | 1.22 | 1.08-1.37 | 1.40 | 1.24-1.59 |
| Liver disease | 2.39 | 1.35-4.23 | 1.64 | 1.01-2.55 | 1.67 | 1.08-2.56 |
| Renal disease | 1.52 | 1.20-1.91 | 1.70 | 1.47-1.98 | 0.99 | 0.84-1.16 |
| Dementia | 1.95 | 1.55-2.46 | 2.31 | 1.98-2.68 | 1.05 | 0.88-1.26 |
| Depression | 1.03 | 0.72-1.47 | 1.02 | 0.81-1.29 | 1.03 | 0.80-1.33 |
| Cancer | 3.93 | 2.97-5.19 | 1.92 | 1.52-2.43 | 1.21 | 0.93-1.57 |
| Coronary heart disease | 1.01 | 0.80-1.42 | 1.42 | 1.22-1.66 | 2.12 | 1.83-2.45 |
| VTE | 1.65 | 1.19-2.30 | 1.75 | 1.39-2.20 | 1.39 | 1.09-1.79 |
| Alcohol | 0.52 | 0.31-0.87 | 0.79 | 0.58-1.07 | 1.00 | 0.74-1.35 |
| Intracraneal haemorrage | 2.79 | 1.97-3.94 | 1.45 | 1.09-1.94 | 0.99 | 0.71-1.39 |
| Gastrointestinal bleeding | 3.56 | 2.26-5.61 | 1.86 | 1.24-2.78 | 0.84 | 0.49-1.45 |
| Other bleeding | 1.08 | 0.86-1.40 | 0.94 | 0.81-1.10 | 1.05 | 0.91-1.22 |
| Length of stay | 1.00 | 1.01-1.01 | 1.00 | 0.99-1.00 | 0.99 | 0.99-0.99 |
